# Pain levels during distraction osteogenesis with lengthening nails in 168 cases

**DOI:** 10.1007/s00590-022-03266-3

**Published:** 2022-04-27

**Authors:** Nikolaus Degen, N. de Almeida Lopes, F. Wolf, J. Fürmetz, E. Euler, W. Böcker, P. H. Thaller

**Affiliations:** 1 Workgroup 3D-Surgery, LMU Munich, Campus Innenstadt: Ziemssenstr. 1, 80336 Munich, Germany; 2 Department of Orthopaedics and Trauma Surgery, Musculoskeletal University Center Munich (MUM), University Hospital, LMU Munich, Campus Innenstadt: Ziemssenstr. 1, 80336 Munich, Germany

**Keywords:** Lengthening nails, distraction osteogenesis, Pain, External fixators, Osteotomy

## Abstract

**Purpose:**

To firstly examine the pain levels during distraction osteogenesis (DO) with lengthening nails (LN) in a large sample.

**Methods:**

A total of 168 cases underwent DO of the tibia or femur with five different models of LN. Under a standardized medical regime, daily pain levels were noted as nominal rating scale (NRS) score (0–10) during the distraction phase. NRS scores and several potential influence factors (LN model, bone, approach, side, age, gender) were evaluated.

**Results:**

The mean distraction length was 39.1 ± 14.4 mm. The average NRS score decreased from postoperative day 1 with 2.84 nonlinearly by 1.03 points (36.3%) over the course of 62 days to an average score of 1.81. The mean decrease during the first thirty days was 0.67(23.6%). Subgroup analysis did not reveal any influence factors.

**Conclusion:**

Pain levels during the distraction phase are overall low, continuously decreasing, and well manageable with mostly non-opioid analgesics.

## Introduction

Distraction osteogenesis (DO) of the long tubular bones is performed in cases of limb length discrepancies due to congenital, developmental and posttraumatic/postinfectious length deficits as well as for stature lengthening due to dysplasia or in rare cases for cosmetic reasons.

The basic principles of DO regarding surgical technique, callus growth, distraction timing and rate were developed using circular external fixation (EF) systems and are still valid today. With EF however, pins and wires unavoidably tether and cut through the soft tissues with progressing distraction, leading to pin site infections, while the frame restricts the range of motion for the surrounding joints [1, 2]. In combination, those factors are likely to cause severe pain. Pain is regarded as the second most common complication in DO using EF after pin-track infections [3]. Early fully implantable lengthening systems (Albizzia nail, Intramedullary Skeletal Kinetic Distractor ISKD^®^) also required patients to do painful rotational movements up to 15× in a row to trigger the nail’s distraction mechanism, which explains the nickname “twist and shout” given to the Albizzia nail. High pain levels reportedly required hospital readmission in more than one third of patients treated with the Albizzia nail and distraction under general or epidural anesthesia in around 25% [4, 5]. The ISKD^®^ nail’s distraction rate was unreliable due to often occurring malfunction of the ratchet mechanism leading to abnormally low or high distraction rates (“runaway nail”), which has been shown to be associated with significantly higher pain levels during distraction compared to regularly functioning nails [6].

EF and early lengthening nails (LN) established the reputation of limb lengthening as painful procedure. Many so-called device-related complications of EF like soft tissue infections and scarring, reduced joint mobility, bone regenerate deformity, late fracture and low patient implant acceptance have been object of several studies emphasizing the reduction of those complications with LN [7–10]. Pain levels, however, have yet been investigated rarely and only for single implants on a recall base or unclear records [2, 6]. With further development of LN toward motorized nails with magnetically and electronically driven distraction mechanisms, nowadays patients’ pain levels are believed to be low during distraction and activity.

The goals of this study were (1) to evaluate pain levels in a large sample with different types of LN based on patients’ daily records and (2) to investigate potential influence factors on pain like the choice of implant, patients’ age/gender, bone/side treated, and surgical approach.

## Patients and methods

### Sample generation and data acquisition

In an institutional review board-approved retrospective single surgeon study, patients’ daily records of pain levels during the distraction period of DO with LN were evaluated.

Between 2003 and 2018, 224 distraction procedures of femur or tibia were performed by a single surgeon (PHT) on 201 patients using 5 different intramedullary systems (listed in order of development) in cases of posttraumatic or developmental limb length difference with or without additional axis or torsional deformity suitable for correction using intramedullary fixation [11]:

- ISKD^®^ (Orthofix, Verona, Italy; roll ratchets, telescoping)
- Phenix^®^ (Phenix medical, Paris, France; magnetic, telescoping)
- Fitbone^®^ (Wittenstein intens, Igersheim, Germany)

- TAA (“telescope active actuator”; electromotive, telescoping)
- SAA (“sliding active actuator”; electromotive, telescoping)
- Precice® (NuVasive, San Diego, USA; magnetic with gears, telescoping).

56 cases (50 patients) were excluded as one or more of the following exclusion criteria applied:

- More than 5% of NRS scores incomplete
- Other basic data missing (distraction distance, LN model, patient data)
- Combined techniques of DO (e.g., lengthening over nail)
- Dysfunctional LN (e.g., implant breakage or uncontrolled distraction rate).

A total of 168 remaining cases (151 patients, 68 women and 83 men) were included in the study. The mean age at surgery was 28.5 ± 12.0 years. All patients kept so-called pain diaries during the distraction phase using a standardized form to document their pain level on a daily base using a nominal rating scale (NRS, score from 0 to 10 with the standardized definition of 0 indicating no pain, 5 for moderate pain, and 10 for worst pain imaginable) to rate the average pain level of the last 24 h and document the distraction distance and frequency and note comments [12]. Patients were asked to rate pain levels each day in private when going to bed, so usually several hours after the last analgesic intake. The NRS score from 0 to 10 is comprehended rapidly by patients, highly sensitive, and its data can be easily used for statistical analysis [13].

The following data from our documentation of each case were added for analysis: patient’s gender and age at time of surgery, bone and side treated, LN used, surgical approach, distraction beginning, end, and pauses, distraction distance and time between surgery and beginning of full weight bearing (bone consolidation).

### Surgical technique

For the femur, an antegrade or retrograde approach is used depending on several factors like patients’ age, joint condition, bone configuration and others [14]. For minimally invasive transarticular approaches to the femur and tibia, custom-made instrumentation is used to impact steel sleeves into the cancellous bone, hereby protecting the soft tissue and preventing entry point migration [15]. On the femur, Schanz screws are set in both the proximal and distal segment for torsional control only and removed after fixation of the LN according to the technique intented for the respective implant. A minimally invasive drill bit corticotomy is performed on the femur or tibia in the meta-diaphyseal region and completed with a chisel, similar to the technique described by De Bastiani et al. [16]. The medullary channel is prepared using rigid reamers, and the drill dust is placed as bone graft into the osteotomy site after insertion of the nail. Blocking screws might be used for fine tuning of the resulting alignment [17]. On the tibia, a complete minimally invasive subcutaneous preventive fasciotomy of the anterior compartment is performed by routine in all patients included in this study, using a fasciotome through the stab incision made for the osteotomy [18]. Acute lengthening of 1 mm is performed to ensure correct function of the implant.

### Postoperative treatment

Except for rare changes due to drug interactions, allergies, weight adaptation or other reasons, all patients were given the same medication consisting typically of pain medication according to the WHO analgesic ladder step 2 (Novaminsulfon 3 × 1 g and Tramal 2 × 50/4 mg during the first two to four days with opioids from then on being administered only on demand during stationary treatment), Vitamin D3 2 × 1000 IE and Calcium 2 × 1000 mg to promote bone healing, and Enoxaparin 40 mg s.c. for deep vein thrombosis prophylaxis during the entire period of incomplete weight bearing. Nonsteroidal anti-inflammatory drugs were not given at all due to their potentially inhibiting effect on bone healing. Regularly, distraction along with pain protocols started on the third postoperative and was performed at a rate of 1 mm/day from then on. Weight bearing of 20 kg on crutches was practiced during physical therapy starting on the first postoperative day and maintained until sufficient bone consolidation was observed after the end of distraction. Hospital discharge followed regularly on the sixth day. Distraction and consolidation progress were monitored on weekly to biweekly clinic visits using focused radiographs of the distraction site with a standardized film-focus distance of 1.15 m, along with joint mobility and other clinical parameters. In the follow-ups during consolidation phase, consolidation was assessed based on both clinical and radiological criteria to determine the beginning of full weight bearing. In all cases, devices were removed about 12 months after consolidation was achieved.

### Statistical methods

The daily NRS pain score and all other variables named before were collected, and the distraction index and consolidation index were calculated. As the NRS scores are a discrete and limited variable, a normal distribution was not to be expected. Univariate Kruskal–Wallis ANOVA was performed to investigate a correlation between the NRS pain score and the following variables considered by the authors to possibly influence the patients’ pain ratings significantly:

- LN model (ISKD^®^, Phenix^®^, Fitbone TAA^®^, Fitbone SAA^®^, Precice^®^)
- Bone treated (femur, tibia)
- Surgical approach at the femur (antegrade, retrograde)
- Patient’s age group (0–20, 21–40, 41–60, 61–80 years)
- Side treated (left, right)
- Patient’s gender (male, female)

**Fig. 1:**
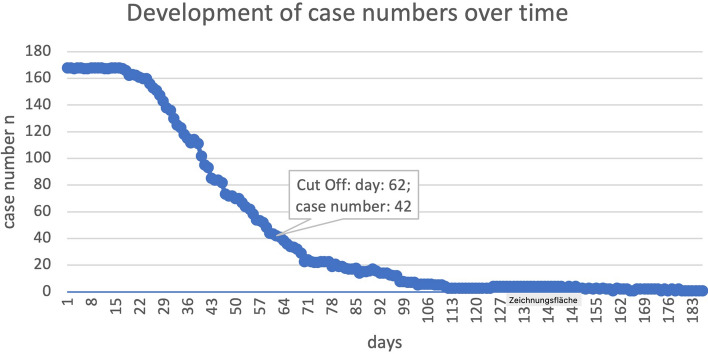
NRS pain scores were evaluated until 25% of the initial case number (*n* = 42) reached at day 62 of distraction

Due to patients’ individual distraction goals and a therefore continuously decreasing case number, the analysis of the NRS pain levels was performed only over the time of a decrease to 25% of the initial 168 cases to ensure a sufficient sample size toward the end of the included NRS scores (42 cases, reached at day 62 after surgery, see Fig. 1).

## Results

**Fig. 2:**
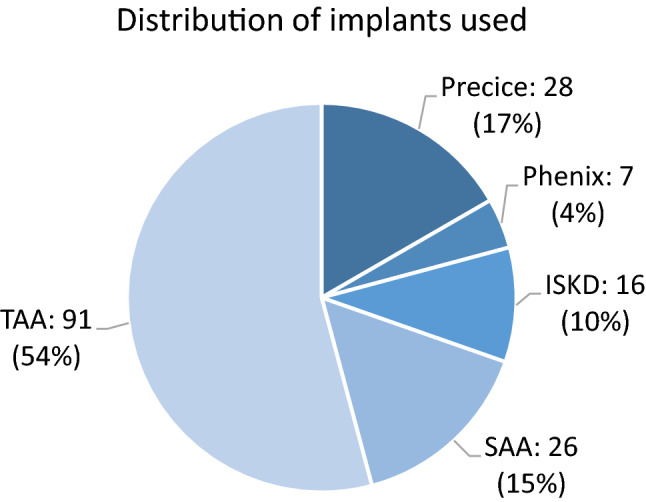
Case numbers of the different distraction nails used

A total of 168 nails were implanted in 151 patients (68 women, 83 men) with 11 patients undergoing 2 distraction treatments of different bones and 6 patients undergoing prolonged distraction treatment of one bone by using the same nail model twice in a row to generate a distraction length of up to 100 mm in one case and 160 mm in another case, the latter with a 12-month interval between treatments. Fitbone^®^ TAA was used 91×, Precice^®^ 28×, Fitbone^®^ SAA 26×, ISKD^®^ 16×, and Phenix^®^ 7× (see Fig. 2).

A total of 113 LN were implanted into the femur, 55 into the tibia. Surgical approach to the femur was retrograde in 61 cases and antegrade in 45 cases with missing data in seven cases. The left side was treated 92× and the right side 76×.

The mean distraction length was 39.1 ± 14.4 mm (37.0; 41.3) during a mean distraction time of 53.6 ± 28.5 days (49.3; 57.8) resulting in a mean distraction index of 0.83 ± 0.32 mm/day (0.78; 0.88) or 14.1 ± 6.5 days/cm (13.3; 15.1).

The mean time between surgery and full weight bearing was 6.4 ± 3.5 months (5.8; 7.0), resulting in a mean consolidation index of 41.2 ± 30.7 days/cm (36.9, 45.4).

In no case, readmission in our clinic for pain control or continuation of the distraction treatment was necessary.

**Fig. 3:**
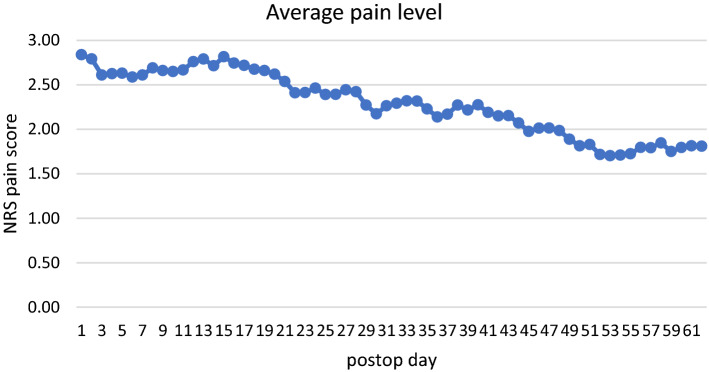
The highest average NRS pain score was found on the first postoperative day

The highest average NRS pain was found on postoperative day 1 with 2.84 and from there on decreased nonlinearly by 1.03 points (36.3%) over the course of 62 days to an average score of 1.81 (see Fig. 3). The mean decrease during the first thirty days was 0.67(23.6%). The mean daily decrease of the score was 1.7 points (0.59%) in total and 0.22(0.79%) during the first thirty days.

**Fig. 4:**
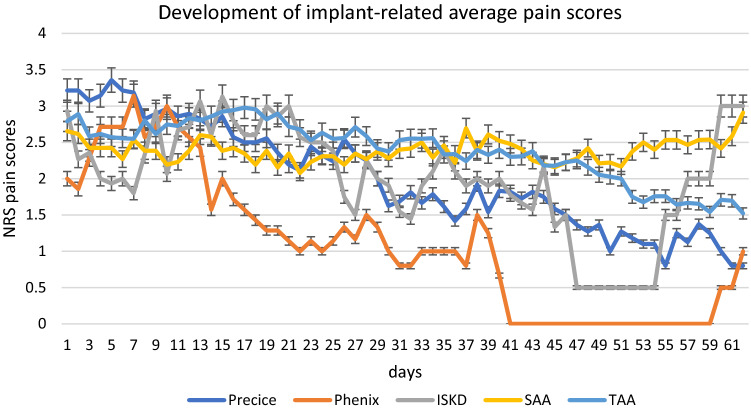
Average pain scores during distraction depending on the implant did not show significant differences (error bars indicate the 95% confidence interval). Patients’ completion of the distraction goal can cause drops to lower pain levels with the remaining patients (Phenix, ISKD)

Subgroup analysis did not reveal any influence of the included parameters on the NRS pain score: No statistical difference depending on the type of implant, gender, bone or side treated, surgical approach to the femur, or the patients’ age group was detected (see Table 1/Fig. 4).

**Table 1:** Average NRS pain scores depending on the variables tested in subgroup analysis

	Mean NRS pain score	*n* (day 1)	*p*-value
Gender: male	2.22	83	> 0.05
Gender: female	2.19	68	
LN: ISKD	2.00	16	> 0.05
LN: phenix	1.09	7	
LN: fitbone TAA	2.39	90	
LN: fitbone SAA	2.39	25	
LN: precice	2.01	28	
Femur: antegrade	2.36	106	> 0.05
Femur: retrograde	2.35	61	
Bone: femur	2.35	113	> 0.05
Bone: tibia	2.17	54	
Age 0–20	2.10	67	> 0.05
Age 21–40	2.47	65	
Age 41–60	2.31	35	
Age 61–80	1.00	1	
Left leg	2.23	91	> 0.05
Right leg	2.36	76

## Discussion

The focus of this study was to investigate the patients’ pain status during the distraction period of a limb lengthening therapy using intramedullary LN in a single surgeon study.

Based on daily documented NRS pain scores of 168 cases over the course of up to 62 postoperative days, our findings reflect an overall low initial average pain level which typically starts at the peak on the first postoperative day and is followed by a continuous nonlinear decrease of 23.6% within the first month under oral mostly non-opioid analgesics. Several treatment parameters hypothesized as possible influencing factors on the pain level could not be confirmed as significant influences (LN model, bone, surgical approach, age, side, gender). Even the early LN (Albizzia, ISKD) were not found to be associated with higher pain scores, as several reports on the pain status of patients treated with these implants might suggest [4–6].

Few other studies report results on the patients’ pain status during the distraction period with LN. They are based on comparably small sample sizes and recall ratings of several weeks to months assessed during follow-up appointments. Lee et al. report a pain score of three on the visual analogue scale (VAS) for 14 regularly distracting ISKD^®^ nails, comparable to our finding of the average initial postoperative NRS pain score [6]. Laubscher et al. report a higher VAS score of 4.4 for the distraction phase with Precice^®^ nails in 20 cases [2].

This study is not without limitations. Quantitative assessment of the highly subjective experience of pain is further complicated by the multifactorial nature of pain generation and perception. Psychosocial factors seem to influence the assessment and also the accuracy of remembering pain [19, 20]. Chronified pain as a separate entity is known to significantly increase retrospective pain ratings [19]. Also, there is no consent on whether the current pain level influences the rating of past pain [21]. Even though in this study the part of patients affected by these factors is expected to be low, it must be stated that they were not specifically assessed.

In all pain ratings that are not solely focused on the distraction process itself but on the distraction period as a section of the entire lengthening treatment, recall bias plays a more or less relevant role. The longer and the more distant the interval to be scored, the greater its potential influence. Recalled pain ratings for entire weeks or months appear to be significantly higher than for 24-h intervals of the same time period [20, 22]. Pain ratings of the last 24 h, however, seem to be well comparable to those of 2-hour intervals [21]. Therefore, in this study, a very low recall influence can be assumed. On the other hand, it is suggested that the act of reporting pain itself could already increase its rating as patients focus more on the pain experience (reporting bias) [23]. This effect is possibly stronger with short-term rating intervals.

LN are known to have several advantages over EF such as reduced device and distraction related complications, significantly lower pain levels for modern nails (compared to monorail EF), and an overall higher patient satisfaction with the entire lengthening treatment [2, 24, 25]. Pain levels in the distraction period are overall low and continuously decreasing, after the first postoperative days pain is well manageable with non-opioid analgesics and independent of a whole range of hypothesized influencing factors. Therefore, patients concerned about the pain of a lengthening treatment with modern distraction nails should be made aware of these benefits.
